# Potential Role of Platelet-Activating C-Type Lectin-Like Proteins in Viper Envenomation Induced Thrombotic Microangiopathy Symptom

**DOI:** 10.3390/toxins12120749

**Published:** 2020-11-27

**Authors:** Chengbo Long, Ming Liu, Huiwen Tian, Ya Li, Feilong Wu, James Mwangi, Qiumin Lu, Tarek Mohamed Abd El-Aziz, Ren Lai, Chuanbin Shen

**Affiliations:** 1 Key Laboratory of Bioactive Peptides of Yunnan Province/Key Laboratory of Animal Models and Human, Disease Mechanisms of Chinese Academy of Sciences, Kunming Institute of Zoology, KIZ/CUHK Joint Laboratory of Bioresources and Molecular Research in Common Diseases, Kunming Institute of Zoology, Chinese Academy of Sciences, Kunming 650223, China; longchengbo@mail.kiz.ac.cn (C.L.); 20B928033@stu.hit.edu.cn (H.T.); wufeilong@mail.kiz.ac.cn (F.W.); mwangij1124@yahoo.com (J.M.); lvqm@mail.kiz.ac.cn (Q.L.); rlai@mail.kiz.ac.cn (R.L.); 2 School of Life Sciences, University of Chinese Academy of Sciences, Beijing 100009, China; 3 Department of Molecular and Cell Biology, School of Life Sciences, University of Science and Technology of China, Hefei 230027, China; qhdming@mail.ustc.edu.cn; 4 Key Laboratory of Laboratory Medicine of Yunnan Province/Department of Clinical Laboratory, the First Affiliated Hospital of Kunming Medical University, Kunming 650032, China; liya@ydyy.cn; 5 Key Laboratory of Cardiovascular Disease of Yunnan Province, Kunming 650051, China; 6 Department of Cellular and Integrative Physiology, University of Texas Health Science Center at San Antonio, San Antonio, TX 78229-3900, USA; mohamedt1@uthscsa.edu; 7 Zoology Department, Faculty of Science, Minia University, El-Minia 61519, Egypt; 8 Sino-African Joint Research Center, CAS, Kunming Institute of Zoology, Kunming 650223, China; 9 Department of Laboratory Medicine, LKSKI-Keenan Research Centre for Biomedical Science, St. Michael’s Hospital, University of Toronto, Toronto, ON M5B 1W8, Canada

**Keywords:** snake venom, C-type lectin-like proteins, platelet, cerebral ischemia, thrombotic microangiopathy

## Abstract

Envenomation by viperid snakes may lead to severe bleeding, consumption coagulopathy, and thrombotic microangiopathy symptoms. The exact etiology or toxins responsible for thrombotic microangiopathy symptoms after snake envenomation remain obscure. Snake C-type lectin-like proteins (snaclecs) are one of the main non-enzymatic protein constituents in viper venoms, of which a majority are considered as modulators of thrombosis and hemostasis. In this study, we demonstrated that two snaclecs (mucetin and stejnulxin), isolated and identified from *Protobothrops mucrosquamatus* and *Trimeresurus stejnegeri* venoms, directly induced platelet degranulation and clot-retraction in vitro, and microvascular thrombosis has been confirmed in various organs in vivo. These snaclecs reduced cerebral blood flow and impaired motor balance and spatial memories in mice, which partially represent the thrombotic microangiopathy symptoms in some snakebite patients. The functional blocking of these snaclecs with antibodies alleviated the viper venom induced platelet activation and thrombotic microangiopathy-like symptoms. Understanding the pathophysiology of thrombotic microangiopathy associated with snake envenoming may lead to emerging therapeutic strategies.

## 1. Introduction

Envenomation and death caused by snakebites represent a significant public health problem worldwide, particularly in tropical and subtropical areas. More than 100,000 people die from snake venom every year worldwide and more people’s lives are endangered. Snakebite has been defined as a neglected tropical disease by the World Health Organization [1,2,3]. Snake venoms are mixtures of toxic and pharmacologically active peptides and proteins [4,5,6,7]. They are weapons for the snakes to immobilize or digest prey, and also act as a defense against competitors and predators [8,9]. Clinical features of snake bite envenoming include, but are not limited to, local pain and tissue damage, neuroparalysis, coagulopathy, and thrombotic microangiopathy (TMA). Envenomation by elapidae snakes may lead to shock and paralysis due to the neurotoxic components, such as the three-finger toxins and phospholipase A2, while viperid snakes may cause hemotoxic effects due to the varied roles of their toxins which include serine proteases, metalloproteases, disintegrins, and snake venom C-type lectins (snaclecs) [1,2,3,4,5,10,11,12].

Venom-induced consumptive coagulopathy (VICC) occurs during the initial stage after snakebite and resolves rapidly without causing systemic microthrombi or end-organ damages, while TMA resulting from snake envenomation is usually characterized by microangiopathic hemolytic anemia, thrombocytopenia, and microthrombosis, which may lead to functional failure of several organs to include brain, lung, liver, kidney and heart [2,13]. However, the etiology of snake envenomation-related TMA remains largely unknown and may occur in a subset of snakebite patients presenting with VICC [13,14,15].

Snaclecs are considered as one of the major non-enzymatic protein constituents in Viperidae venom [16,17,18,19,20], but the significance of snaclecs for snakebite injuries has not been fully elucidated yet. *Protobothrops mucrosquamatus* (*P. mucrosquamatus*) and *Trimeresurus stejnegeri* (*T. stejnegeri*) belong to Viperidae family which inhibit South and Southeast Asia, and they are a common cause of snakebites [21,22,23]. Our previous studies revealed two typical C-type lectin-like proteins, namely mucetin and stejnulxin, from the venoms of *P. mucrosquamatus* and *T. stejnegeri*, which showed strong abilities to induce platelet aggregation [9,24,25]. Whether these snaclecs contribute to TMA symptom needs to be investigated.

In this study, we demonstrate that mucetin and stejnulxin induce TMA-like symptom characterized by multi-organ injuries and bleeding disorders with an acute consumption coagulopathy syndrome in a platelet-related way. This study fills the knowledge gap by demonstrating that the platelet-activating snaclecs might be the key toxins responsible for snakebite related TMA.

## 2. Results

### 2.1. Mucetin and Stejnulxin Induce Microthrombosis in Multiple Organ Tissues and Blood Biochemical Alterations in Mice 

**Figure 1:**
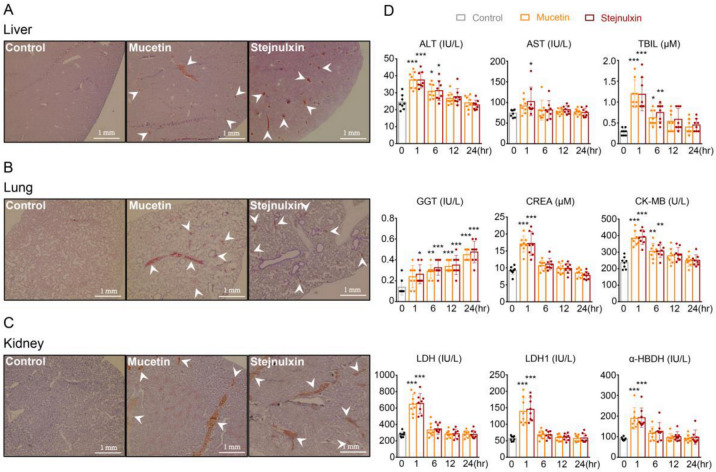
Histological analysis and blood biochemical alterations after the injection of mucetin and stejnulxin in mice. (**A**–**C**) Mice were intravenously injected with normal saline (control), mucetin (30 μg/kg) or stejnulxin (30 μg/kg). Perfused organ tissues from liver (**A**), lung (**B**) and kidney (**C**) were collected 30 min after the injection and processed for hematoxylin and eosin staining. (**D**) Blood alanine aminotransferase (ALT), aspartate aminotransferase (AST), total bilirubin (TBIL), γ-glutamyl transpeptidase (GGT), creatinine (CREA), creatine Kinase-MB subform (CK-MB), lactate dehydrogenase (LDH), lactate dehydrogenase isoenzyme 1 (LDH1) and α-hydroxybutyrate dehydrogenase (α-HBDH) levels were analyzed at different times after the injection. * *p* < 0.05, **p < 0.01, ***p < 0.001.

Viper snake envenoming may lead to thrombosis, emboli and damage routinely in multiple organs [12,13]. To investigate whether mucetin and stejnulxin induce thrombus formation and tissue damage in vivo, we examined the tissue sections with hematoxylin and eosin staining, and evaluated the alteration of blood biochemical parameters which reflects TMA-related tissue damage in mice [26]. Mucetin and stejnulxin have been isolated and identified from *P. mucrosquamatus* and *T. stejnegeri* venoms as previously described [9]. Hematoxylin and eosin staining showed extensive thrombus deposition in small and medium size vasculature of the liver (Figure 1A), lung (Figure 1B), and kidney (Figure 1C) after the injection of mucetin or stejnulxin at 30 μg/kg. Blood alanine aminotransferase (ALT), aspartate aminotransferase (AST), total bilirubin (TBIL), creatinine (CREA), creatine Kinase-MB subform (CK-MB), lactate dehydrogenase (LDH), lactate dehydrogenase isoenzyme 1 (LDH1), and α-hydroxybutyrate dehydrogenase (α-HBDH) were all elevated rapidly 1 h after the injection, and gradually returned to normal 12–24 h later. However, γ-glutamyl transpeptidase (GGT) continued to rise within 24 h (Figure 1D), indicating these snaclecs may lead to both rapid and persistent organ injuries.

### 2.2. Mucetin and Stejnulxin Induce Cerebral Ischemia and Neurological Deficits in Mice

**Figure 2:**
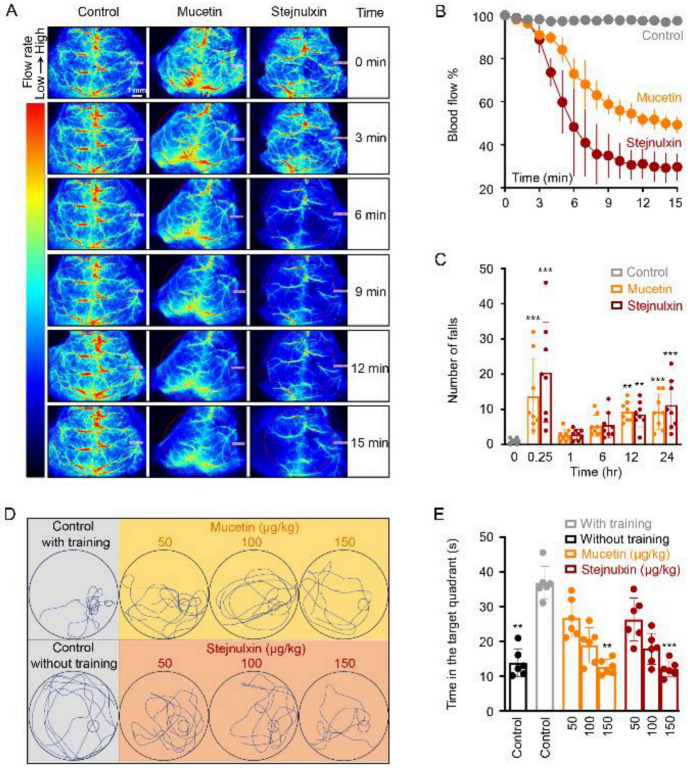
Effect of mucetin and stejnulxin on cerebral blood flow and neurological abnormality of mice. (**A**) Representative images and (**B**) blood flow quantification of mice treated with saline (control), or mucetin and stejnulxin at the concentration of 50 μg/kg. (**C**) Rotarod test was carried out with a speed of 8 rpm to assess the motor function and coordination of mice after the injection. (**D**) Spatial memory was evaluated by Morris water maze test 24 h after the injection, and the distinct swimming paths were recorded during the probe trial. (**E**) Statistical analysis of the time spent in the target quadrant during Morris water maze test. ** *p* < 0.01, ***p < 0.001.

Despite acute cerebral infarction or stroke after snakebites are rare, there were many cases reported after viper envenomation, including the case caused by *T. stejnegeri* [27,28,29,30,31,32,33]. Based on our previous studies, tail vein injection of mucetin or stejnulxin at 50 μg/kg potently reduced the blood flow in cerebral cortex (Figure 2A), the average flow rate decreased to 60.5% and 43.0% of the control group (normal saline) within 15 min (Figure 2A,B). Cerebral hypoxia after infarction or stroke is one of the main reasons for neurological deficits [34]. Rotarod tests showed impaired motor coordination and body balance in mice receiving mucetin and stejnulxin for 15 min, with the average fall times significantly increased from 1 to 13.8 and 20.5, respectively (Figure 2C). However, the fall times decreased sharply 1 h after the injection, but increased again over time, and reached 9.4 and 11.3 on average on the second day (Figure 2C). Spatial memory was also affected by the snaclecs as evaluated by a Morris water maze test (Figure 2D,E). Time spent in the target quadrant was markedly increased from 14.23 to 37.17 s after a seven-day training, while mice spent less time in the target quadrant 24 h after the administration of mucetin or stejnulxin (Figure 2D,E). These suggest that mucetin and stejnulxin may induce a rapid but also persistent effect on neurological deficits.

### 2.3. Mucetin and Stejnulxin Affect Blood Coagulation and Bleeding in Mice.

**Figure 3:**
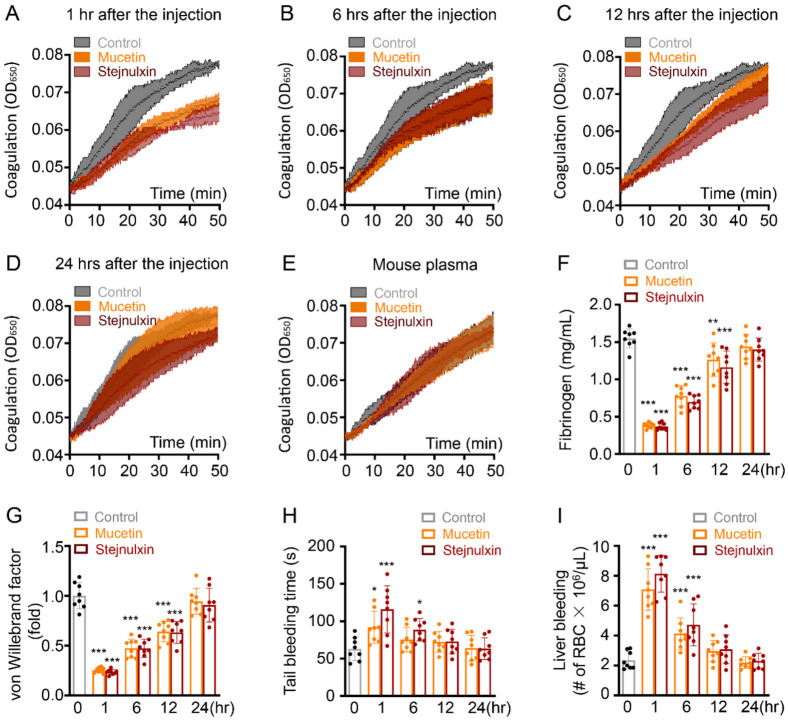
Mucetin and stejnulxin affect mice blood coagulation and increase bleeding risk. Mice were intravenously injected with normal saline (control), 30 μg/kg mucetin or stejnulxin. The plasma of the snaclecs treated mice was collected 1 (**A**), 6 (**B**), 12 (**C**), 24 (**D**) hours after the injection with sodium citrate anticoagulant tubes. The clotting process (50 min) of each plasma sample was initiated by 10 mM CaCl_2_ and monitored by microplate reader at the absorbance of 650 nm. (**E**) Mice plasma was collected without treatment, the clotting process (50 min) was detected with or without mucetin or stejnulxin (2 μg/mL) in vitro. (**F**) Fibrinogen and (**G**) von Willebrand factor changes were detected in citrated plasma from mice receiving normal saline (control), mucetin or stejnulxin at 30 μg/kg. (**H**) Mice tail bleeding time and (**I**) erythrocyte count in peritoneal lavage fluid after a liver operation were measured with or without mucetin or stejnulxin administration at 30 μg/kg for different time periods. * *p* < 0.05, ** *p* < 0.01, *** *p* < 0.001.

VICC is one of the major symptoms after viper envenomation, which is usually only complicated by bleeding without organ damage and recovers rapidly [12]. To investigate whether mucetin and stejnulxin have an effect on coagulopathy, we evaluated mice plasma recalcification time, fibrinogen concentration, von Willebrand factor (VWF) content changes and bleeding disorders after the injection. Mucetin and stejnulxin at 30 μg/kg reduced mice plasma coagulation in vivo, but this effect rapidly recovered within 24 h (Figure 3A–D). However, both of the snaclecs at 2 μg/mL had no effect on recalcification time with citrated mouse plasma in vitro (Figure 3E), suggesting these toxins did not directly target the coagulation pathway. Consistent with the recalcification assay, the content of plasma fibrinogen and VWF rapidly decreased but recovered within 24 h after the injection (Figure 3F,G). Mucetin and stejnulxin also transiently increased the bleeding in both tail and liver (Figure 3H,I). These suggest that mucetin and stejnulxin may lead to transient and rapidly recovering coagulopathy.

### 2.4. Mucetin and Stejnulxin Promote Platelet Activation and Clot Retraction.

**Figure 4:**
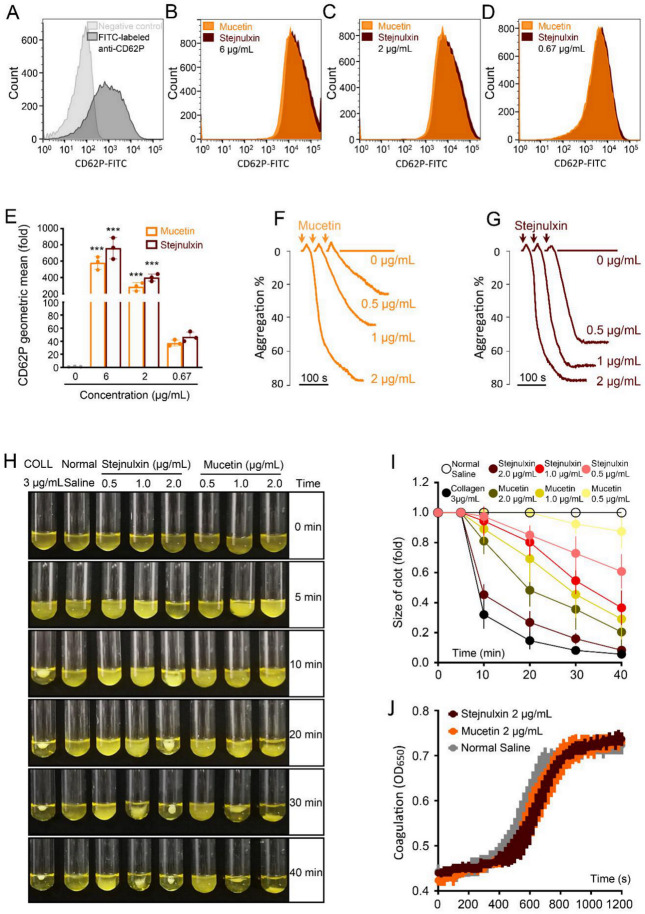
Effect of mucetin and stejnulxin on human platelet activation and platelet-rich plasma clot retraction. Binding of FITC-labeled anti-CD62P antibody to washed platelets in the absence (**A**) or presence of 6 (**B**), 2 (**C**), 0.67 (**D**) μg/mL mucetin and stejnulxin were detected by flow cytometry. (**E**) The fold change of the CD62P on platelet membrane between mucetin or stejnulxin treated groups and the non-treated group (normalized to 1). (**F**,**G**) Mucetin and stejnulxin induced human platelet aggregation in a dose-dependent manner. The Y axis represents the aggregation ratio (%) detected by aggregometer and the horizontal bar represents the time scale. (**H**) Effects of mucetin and stejnulxin on clot retraction of human PRP in the presence of 10 mM CaCl_2_, with the collagen (COLL) as positive control. (**I**) Statistical analysis of mucetin and stejnulxin induced platelet clot retraction over a 40-min time period. (**J**) Effect of mucetin and stejnulxin on human plasma clotting in the presence of 10 mM CaCl_2_. All data are expressed as mean ± SD from 3 experiments. *** *p* < 0.001.

Despite that some snaclecs inhibit platelet function [35,36,37], many proteins of this family have been reported to activate platelets, including mucetin and stejnulxin [24,25,35]. P-selectin (CD62P) is a transmembrane protein stored in the alpha granules of platelet, which will be transferred onto the membrane surface and plasma with the activation of platelets [38]. The degranulation of P-selectin is also associated with TMA symptoms [38]. Mucetin and stejnulxin induced platelet degranulation potently in a dose-dependent manner in human platelets (Figure 4A–E). Mucetin and stejnulxin at 6 μg/mL significantly increased membrane P-selectin levels by 579 and 758 folds (Figure 4B) on average compared to control (Figure 4A). Both the washed platelets [24,25] and platelets in human plasma (Figure 4F,G) could be aggregated by the snaclecs in a dose-dependent manner in the aggregometer. Platelet-driven clot retraction plays important roles in bleeding and thrombotic disorders [39]. Mucetin or stejnulxin initiated clot retraction in human platelet-rich plasma (Figure 4H,I), despite the fact that they had no effect on human plasma coagulation in vitro (Figure 4J).

### 2.5. Blocking Mucetin and Stejnulxin by Antibody Alleviate Crude Venom Induced TMA and Bleeding Disorders.

**Figure 5:**
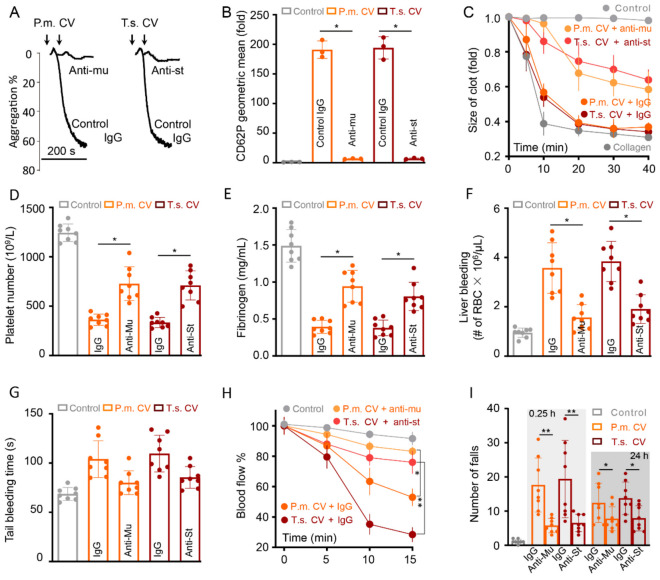
Functional blocking of mucetin and stejnulxin in viper venoms alleviate crude venom induced platelet activation and TMA symptoms in mice. Antibody against mucetin (Anti-mu) or stejnulxin (Anti-st) at 50 μg/mL functionally inhibited human platelets aggregation (**A**), secretion (**B**) and clot retraction (**C**) induced by crude venoms from *P. mucrosquamatus* (P.m. CV) or *T. stejnegeri* (T.m. CV) at 1 μg/mL. Anti-mu (100 μg/mice) and Anti-st (150 μg/mice) ameliorated thrombocytopenia (**D**), elevated fibrinogen concentration (**E**), and alleviated bleeding disorders (**F**,**G**) after a single injection of crude venoms at 100 μg/kg. Anti-mu (100 μg/mice) and Anti-st (150 μg/mice) alleviated cerebral ischemia (**H**) and neurological deficit induced by the crude snake venoms at 100 μg/kg (**I**). * *p* < 0.05, **p < 0.01.

To clarify the role of snaclecs in snake venom induced TMA symptom, we used purified polyclonal antibodies (Supplementary Figure S1) to neutralize mucetin or stejnulxin in crude venoms. Crude venoms from *P. mucrosquamatus* and *T. stejnegeri* at 1 μg/mL significantly induced platelet aggregation (Figure 5A), degranulation (Figure 5B) as well as clot retraction (Figure 5C and Supplementary Figure S2) in vitro. However, antibodies against mucetin and stejnulxin significantly blocked the venom induced platelets activation (Figure 5A–C). In vivo analysis showed an obvious decrease of platelet numbers (Figure 5D) and fibrinogen concentration (Figure 5E) as well as abnormal hemostasis (Figure 5F,G) after the injection of viper venoms at 100 μg/kg, while antibodies against mucetin and stejnulxin showed a protective role (Figure 5D–G). These antibodies also significantly alleviated the viper venom induced cerebral infarction and neurological deficits as detected by blood flow imaging system (Figure 5H and Supplementary Figure S3) and rotarod test (Figure 5I). This means that snaclecs, including mucetin and stejnulxin, may play important roles in snake envenomation induced coagulopathy and TMA-like symptoms, and functional blocking antibodies against these snaclecs may alleviate these pathological changes induced by snakebites.

## 3. Discussion

Cobra venom mainly contains neurotoxins, while venoms from Viperidae mostly have hemorrhagic toxins, always inducing coagulopathy and platelet dysfunction, which thus may lead to bleeding disorders and multi-organ injuries [1,2,3,13]. Here, we show that two snaclecs from two viper venoms induce TMA-like symptoms with transient and rapidly recovering VICC in a platelet-related way. These snaclecs may contribute to viper envenomation-induced TMA symptoms in human.

Animal venoms are highly evolved chemical weapons for efficient defense and predation [9,40,41]. Snacles are rich in snake venoms and considered as one of the major non-enzymatic proteins in viperid venoms [6,16,17,18,19,20]. They typically fold like C-type lectins, such as selectins and mannose-binding proteins, without containing the classic calcium/sugar-binding loop. Structurally, snaclecs are usually composed of heterodimeric α and β subunits, which may form larger complexes via oligomerization. Many snaclecs have been proved to interact with varieties of proteins on platelet such as GPIb, GPVI and integrins, which are considered as critical regulators for platelet activation, thrombosis, and hemostasis [35,42]. So far, whether snaclecs lead to VICC or TMA has not been elucidated yet. 

VICC is one of the most important effects of snakebites [43,44,45]. However, clinical features more consistent with TMA have been reported in recent years after viper envenomation [13,46,47,48,49]. Snake envenomation associated TMA usually arises in conjunction with VICC, and was thought to result from VICC to some extent, but the latter results from coagulation pathway activation mediated by toxins such as factor X activators, thrombin-like enzymes and prothrombin activators, without causing systemic microthrombi or end-organ damages and resolves rapidly [13,46,47,48]. However, TMA is featured by intraluminal platelet thrombosis and multi-organ damages. To date, the exact etiology and toxins responsible for TMA after snakebites remain largely unknown [13,14,48]. Here, we found mucetin and stejnulxin indeed led to transient and rapidly recovering coagulopathy (Figure 3), which may represent the VICC symptom in human. However, injection of these snaclecs induced microthrombi in various organ tissues (Figure 1A–C) complicated with rapid multi-organ injury of liver, kidney and heart, as indicated by blood biochemical alterations like ALT, TBIL, CREA, and LDH in mice (Figure 1D). Blood GGT which reflects the progress of liver disease and metabolic syndrome [50,51] continued to rise within 24 h after the injection (Figure 1D). Meanwhile, rotarod test and Morris water maze task showed that the snaclecs not only lead to a rapid but also a persistent effect on neurological deficits resulted from cerebral ischemia (Figure 2), which may represent the viper envenomation-induced cerebral ischemia symptoms in humans [27,28,29,30,31,32,33]. The activated platelets dramatically increase cell-based thrombin generation and coagulation via secreting coagulation factors and providing negatively-charged surfaces [52,53]. Considering the lack of enzymatic and procoagulant activity of the snaclecs (Figure 3E and Figure 4G), we think that mucetin and stejnulxin may lead to coagulopathy indirectly and might be one of the key reasons that responsible for the different, but partially overlapping clinical symptoms of consumptive coagulopathy and TMA. Different species of venomous snakes or the same species from different habitats may have different amount/proportion of coagulation activators and snaclecs [3,20]. Based on our study, it is possible that the venoms rich in platelet-activating snaclecs may cause more severe TMA symptoms. The correlation between platelet-activating snaclecs level and TMA merits future investigation in a clinical context and will help us to better understand and distinguish the venom-induced consumptive coagulopathy and TMA, which may further benefit the diagnosis, prevention, and treatment of TMA induced by snake envenomation. 

TMA, or more specifically, thrombotic thrombocytopenic purpura (TTP) and hemolytic uremic syndrome (HUS), is characterized by intraluminal platelet-rich thrombi, thrombocytopenia and organ infarction, which mainly results from deficiency of ADAMTS13, the protease cleaves von Willebrand, thus leading to the accumulation of ultra-large von Willebrand factor (ULVWF) multimers and the formation of platelet thrombus in human [54,55,56]. Therefore, platelet is crucially important in TMA occurrence. Mucetin and stejnulxin are thought to be platelet activators via acting on GPIbα [24,57,58] and GPVI [25], respectively, and no other receptors or cells have been discovered as a target of these snaclecs up to now. These snaclecs induced platelet degranulation represented by P-selectin (Figure 4A–E) and initiated clot retraction driven by platelets (Figure 4H,I). P-selectin is an adhesion molecule found in storage granules of platelets and endothelial cells. It will be expressed on the cell surface and also secreted into the plasma upon activation of the cells. P-selectin mediates the adhesion of platelets and monocytes on endothelium, and may promote TMA by anchoring ULVWF multimers on endothelial cell surfaces [38,59]. Mucetin and stejnulxin indeed reduced plasma VWF levels rapidly (Figure 3G), suggesting that VWF anchoring is engaged in the snaclecs-induced TMA-like symptom. Platelet-driven clot retraction plays an important role in thrombotic disorders [39], which inhibits clot lysis as a consequence of decreased access of fibrinolytic proteins [60], thus facilitates the deposition of intracapillary platelet thrombi or fibrin in multiple organs. Therefore, platelet activation may play important roles in mucetin or stejnulxin induced TMA-like symptom. However, snaclecs are functionally versatile and some of the proteins from this family can act on other blood or endothelial cells [20,61]. This study could not completely exclude that mucetin and stejnulxin or some other platelet-activating snaclecs may also interact with the cells except for platelet, such as endothelial cell, which may synergistically contribute to the thrombosis and TMA symptoms. We found the viper venom induced TMA-like symptom was more consistent with TTP rather than HUS in mice, as the profound neurological deficits were detected (Figure 2), while the hemolytic anemia was not significant (data not shown). Functional antibodies against mucetin or stejnulxin significantly alleviated crude viper venom induced VICC and neurological deficits (Figure 5), which further confirms the important role of platelet-activating snaclecs in snake envenomation-induced TMA-like symptoms.

In conclusion, platelet-activating snaclecs, such as mucetin and stejnulxin, are potential factors for viper envenomation-induced TMA symptoms. Functionally blocking of these snaclecs might be a promising strategy to alleviate the viper venom-induced platelet activation and TMA symptoms.

## 4. Materials and Method

### 4.1. Tissue Preparation and Hematoxylin and Eosin (H&E) Staining

Mucetin and stejnulxin were purified as previously reported [9,25,57]. BALB/c mice (6-week-old) of either sex were anesthetized under anesthesia respirator (R540IP, RWD Life Science) as the manufacturer’s instructions, and perfused with normal saline 30 min after tail vein injection of mucetin and stejnulxin. Liver, lung and kidney were fixed with 4% formalin, dehydrated by 40% sucrose solution and then cut into 10-µm-thick sections on a freezing microtome (CryoStar NX50 OP, Thermofisher, Waltham, MA, USA). The sections were subsequently stained with hematoxylin and eosin for further analysis as previously reported [62]. Animal protocol in this work, SMKX2017026, was reviewed and approved by the Animal Care and Use Committee at Kunming Institute of Zoology, Chinese Academy of Sciences in December 2017.

### 4.2. Real-Time Measurement of Cerebral Cortex Blood Flow

The cerebral cortex blood flow of mice was measured as previously reported by Tian H. et al. [9]. Briefly, blood flow in the cerebral cortex of mice (BALB/c, 6-week-old, either sex) was monitored after scalp avulsion by laser-speckle imaging system (RFLSI Pro, RWD Life Science, Shenzhen, China) under an anesthesia respirator (R540IP, RWD Life Science). The real-time blood flow was monitored before and after tail vein injection of mucetin and stejnulxin.

### 4.3. Rotarod Test

Motor coordination and balance was evaluated by rotarod test as previously described [63]. Briefly, before the rotarod test, BALB/c mice (6-week-old) of either sex were trained for 2 days with three consecutive trials every day (5 min per trial). After the intravenous injection of the mucetin and stejnulxin, mice were placed onto motorized rod (LE8500, Panlab, Barcelona Spain) with a speed of 8 rpm, the fall-off times within 3 min on every trial were recorded and analyzed. 

### 4.4. Morris Water Maze

Morris water maze was used to assess the spatial memory of mice as previously reported [64]. Briefly, BALB/c mice (6-week-old) of either sex were trained to find the fixed hidden platform in the water from each of the four quadrants within the maze apparatus before the experiment. Then the platform was removed and the trained mice were allowed to explore freely for 60 s to examine their spatial memory 24 h after the intravenous injection of mucetin and stejnulxin or the crude venoms. Swimming traces were automatically recorded and analyzed by with Morris water maze video analysis system (ZS-001, ZS Dichuang Technological Development Co., Ltd., Beijing, China).

### 4.5. Platelet Aggregation Assay

Platelet-rich plasma (PRP) from healthy donors was obtained from Yunnan Kunming Blood Center and the aggregation assay was performed as we reported recently [65]. Briefly, PRP was placed into an aggregometer (LBY-NJ4, Techlink, Beijing, China) and the aggregation was induced by the addition of mucetin or stejnulxin of different concentrations. 

### 4.6. Membrane P-Selectin Detection

Platelets P-selectin detection was performed as previously described with modifications [66,67]. Briefly, platelet were pelleted from PRP by centrifugation at 500 g for 5 min at room temperature. Then platelets were suspended in Tyrode’s buffer A. Washed platelets were resuspended in Tyrode’s buffer B and incubated with FITC labeled anti-human P-Selectin (CD62P, 304904, Biolegend) with or without mucetin or stejnulxin at 37 °C for 40 min, then subjected to a LSR Fortessa flow cytometer (BD, Franklin Lakes, NJ, USA) for further analysis.

### 4.7. Clot Retraction

A clot retraction assay was performed as we previously described [67]. Briefly, human PRP was incubated with mucetin or stejnulxin at corresponding concentration at 37 °C for 5 min. CaCl_2_ (10 mM) was added to initiate the coagulation at 37 °C. The clots were photographed at different time points. Sizes of the clots were quantified using Image J 1.35 h software.

### 4.8. Plasma Coagulation Assay

Plasma coagulation was assessed by recalcification time as reported previously with some modifications [68]. Briefly, 20 µL of human or mouse platelet-poor plasma (PPP) was dispensed into flat bottom Strip-well microplates (42592, Corning), then HEPES buffer (80 µL, pH 7.4) with or without mucetin or stejnulxin were added into the wells and incubated for 5 min. CaCl_2_ (0.025 M, 50 µL) was added to initiate the coagulation responses. The clotting process was monitored with Microplate reader (BioTek Instrument, Inc., Winooski, VT, USA) by measuring the turbidity of solution at the absorbance of 650 nm.

### 4.9. Bleeding Assay

Tail-tip transection (5 mm) was made to evaluate tail bleeding times with or without mucetin or stejnulxin injection, while a calibrated piece (5.21 ± 0.46 mg) was chopped off from the liver lobe to evaluate liver bleeding as previously described [67].

### 4.10. Generation and Purification of Polyclonal Antibodies against Mucetin and Stejnulxin

Rabbit polyclonal antibodies against mucetin and stejnulxin were produced as previously described [69]. Briefly, animals were primed by subcutaneous multi-point injection of 400 μg of mucetin or stejnulxin in complete Freund’s adjuvant (1 mL, F-5881, Sigma, St. Louis, MO, USA ) on day 0, followed by injections of the half dose of the antigens in incomplete Freund’s adjuvant (F-5506, Sigma) on day 14, 28, and 42. Polyclonal IgG antibodies were then purified from rabbit serum by protein A column (HiTrapTM rProtein A FF, Amersham Biosciences, England) with binding buffer (0.02 M sodium phosphate, pH 7.2) in FPLC system (ClearFirst-3000, Shanghai Flash Spectrum Biotechnology, Shanghai, China), and eluted by 0.1 M sodium citrate buffer (PH 3.0). The eluents were neutralized with 1 M Tris-HCl buffer (PH 9.0) and ultra-filtrated with phosphate buffered saline (PBS) (0.01 M, PH 7.4) for further use. 

### 4.11. Statistical Analysi

Data analysis was performed with GraphPad Prism 8 software, and statistical analysis was performed using nonparametric test with a Dunn’s multiple comparison test. The results were presented as mean ± SD. Significance was defined as * *p* <0.05, ** *p* < 0.01, *** *p* < 0.001.

## Supplementary Materials

The following are available online at https://www.mdpi.com/2072-6651/12/12/749/s1, Figure S1: Purification, titer and function analysis of polyclonal antibody against mucetin and stejnulxin. Figure S2: Purified polyclonal antibody against mucetin and stejnulxin inhibited the clot retraction induced by the crude venoms of *P. mucrosquamatus* and *T. stejnegeri*. Figure S3: Purified polyclonal antibody against mucetin and stejnulxin inhibited the cerebral ischemiathe induced by the crude venoms of *P. mucrosquamatus* and *T. stejnegeri*.
