# Supplementary material for: Potential Role of Platelet-Activating C-Type Lectin-Like Proteins in Viper Envenomation Induced Thrombotic Microangiopathy Symptom
Source: Toxins (Basel). 2020 Nov 27;12(12):749. doi: 10.3390/toxins12120749 (PMC7760373; doi:10.3390/toxins12120749)
Supplement: Supplementary file 1 [file toxins-12-00749-s001.pdf]

Supplementary Materials: Potential Role of Platelet-Activating C-Type Lectin-Like Proteins in Viper Envenomation Induced Thrombotic Microangiopathy Symptom

Chengbo Long, Ming Liu, Huiwen Tian, Ya Li, Feilong Wu, James Mwangi, Qiumin Lu, Tarek Mohamed Abd El-Aziz , Ren Lai and Chuanbin Shen

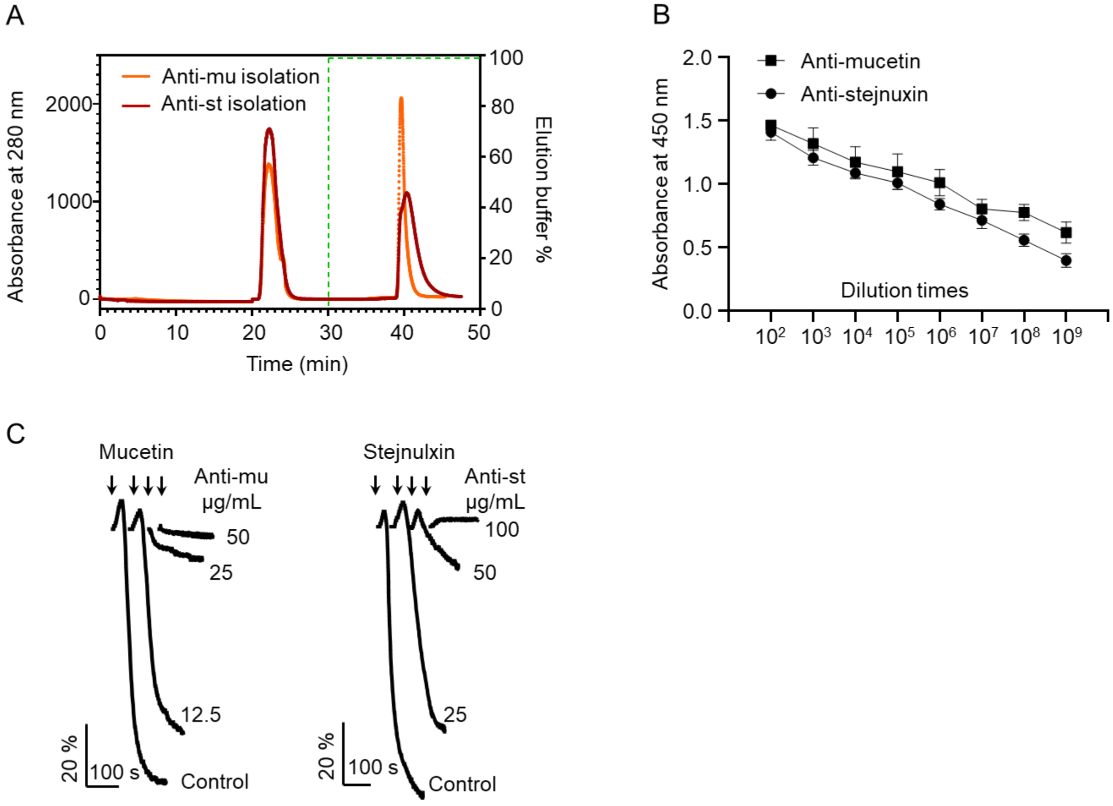

Figure S1. Purification, titer and function analysis of polyclonal antibody against mucetin and stejnulxin. (**A**) Purifucation of IgG from the serum of rabbits immuned with mucetin or stejnulxin. (**B**) The titer of polyclonal antibodies were detected by enzyme-linked immunosorbent assay (ELISA). (**C**) Effect of the purified antibodies against 1 µg/mL mucetin or stejnulxin induced human platelet aggregation.

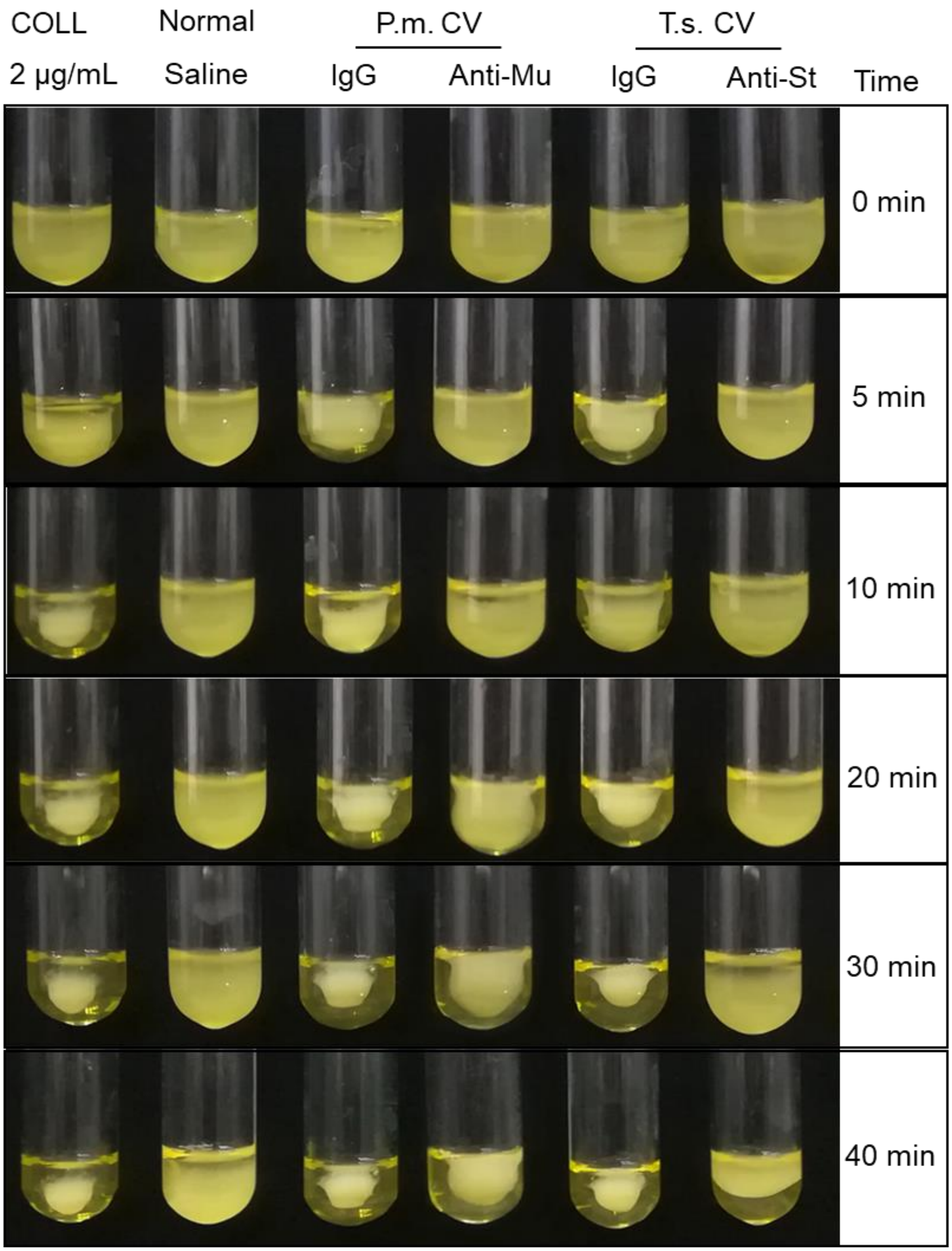

Figure S2. Purified polyclonal antibody against mucetin and stejnulxin inhibited the clot retraction induced by the crude venoms of *P. mucrosquamatus* and *T. stejnegeri*.

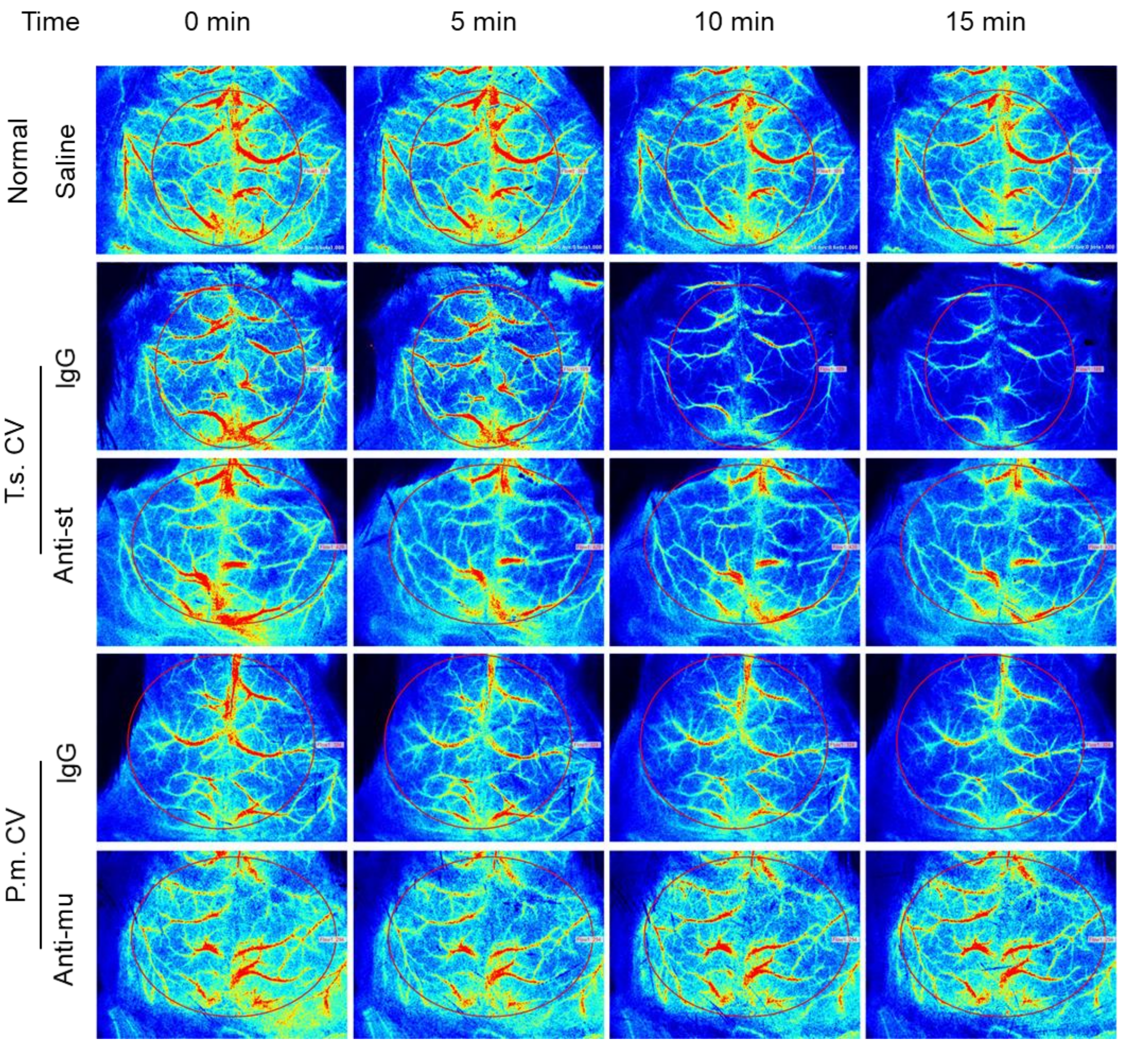

Figure S3. Purified polyclonal antibody against mucetin and stejnulxin inhibited the cerebral ischemia induced by the crude venoms of *P. mucrosquamatus* and *T. stejnegeri*.
